# *α*-Amylase and *α*-Glucosidase Inhibitory Activities of Chemical Constituents from *Wedelia chinensis* (Osbeck.) Merr. Leaves

**DOI:** 10.1155/2018/2794904

**Published:** 2018-05-09

**Authors:** Nguyen Phuong Thao, Pham Thanh Binh, Nguyen Thi Luyen, Ta Manh Hung, Nguyen Hai Dang, Nguyen Tien Dat

**Affiliations:** ^1^Advanced Center for Bio-Organic Chemistry, Institute of Marine Biochemistry (IMBC), Vietnam Academy of Science and Technology (VAST), 18 Hoang Quoc Viet, Caugiay, Hanoi, Vietnam; ^2^Graduate University of Science and Technology, VAST, 18 Hoang Quoc Viet, Caugiay, Hanoi, Vietnam; ^3^National Institute of Drug Quality Control (NIDQC), 48 Hai Ba Trung, Hoankiem, Hanoi, Vietnam; ^4^Center for Research and Technology Transfer, VAST, 18 Hoang Quoc Viet, Caugiay, Hanoi, Vietnam

## Abstract

As part of an ongoing search for new natural products from medicinal plants to treat type 2 diabetes, two new compounds, a megastigmane sesquiterpenoid sulfonic acid (**1**) and a new cyclohexylethanoid derivative (**2**), and seven related known compounds (**3–9**) were isolated from the leaves of *Wedelia chinensis* (Osbeck.) Merr. The structures of the compounds were conducted via interpretation of their spectroscopic data (1D and 2D NMR, IR, and MS), and the absolute configurations of compound **1** were determined by the modified Mosher's method. The MeOH extract of *W. chinensis* was found to inhibit *α*-amylase and *α*-glucosidase inhibitory activities as well as by the compounds isolated from this extract. Furthermore, compound **7** showed the strongest effect with IC_50_ values of 112.8 ± 15.1 *μ*g/mL (against *α*-amylase) and 785.9 ± 12.7 *μ*g/mL (against *α*-glucosidase). Compounds **1**, **8**, and **9** showed moderate *α*-amylase and *α*-glucosidase inhibitory effects. Other compounds showed weak or did not show any effect on both enzymes. The results suggested that the antidiabetic properties from the leaves of *W. chinensis* are not simply a result of each isolated compound but are due to other components such as the accessibility of polyphenolic groups to *α*-amylase and *α*-glucosidase activities.

## 1. Introduction

In recent years, the number of diabetic patients is rapidly rising in most parts of the world, especially in developing Southeast Asian countries [1, 2]. The control of blood glucose concentrations near the normal range is mainly based on the use of oral hypoglycaemic/antihyperglycaemic agents and insulin. However, all of these treatments have limited efficacy and are associated with undesirable side effects [3, 4], leading to increasing interest in the use of medicinal plants for the alternative management of type 2 diabetes mellitus. An effective suggestion for type 2 diabetes management is the inhibition of *α*-glucosidase and *α*-amylase [5].

Natural health-care products derived from medicinal plants or herbs have been developed as alternative or complementary treatments for many common disorders [6]. Several medicinal plants have been the useful sources of novel biologically active compounds. Many pharmaceutical agents have been discovered by screening natural products from plants, many of which have been developed as new leads for pharmaceuticals [7]. Predominantly herbal drugs have been widely used globally for diabetic treatment over thousands of years due to their traditional acceptability and lesser side effects. Therefore, screening of *α*-amylase (1,4-*α*-D-glucan glucanohydrolases; EC. 3.2.1.1) and *α*-glucosidase (*α*-D-glucoside glucohydrolase, EC 3.2.1.20) inhibitors in medicinal plants has received much attention [6, 7].

Among the traditionally used important medicinal plants, the genus *Wedelia* (Asteraceae) contains approximately 107 species in the world, and among them, about 6 species are in Vietnam. They are all herbal plants and distributed in tropical and subtropical regions of Asia and Pacific Islands [8]. *Wedelia chinensis* (Osbeck.) Merr. (Asteraceae) is a deciduous shrub, widely distributed in several Asian countries such as China, Japan, and mainland Vietnam. The leaves, stems, and fruits of this species have been traditionally used in fold medicine for the treatment of chin cough, diarrhoea, diphtheria, faucitis, hemorrhoids, and injuries due to falls, jaundice, and pertussis and are often consumed as tea in the form of infusion [9]. Phytochemically, up to date, a number of secondary metabolites have been identified, including various types of compounds belonging to chemical classes of flavonoids, diterpenoids, and triterpenoids. Besides, several other compounds including common saponins and phytosterol derivatives were also reported in the species, but they appear to have a more limited distribution. Recently, several studies have demonstrated the exploration of pharmacological potential, such as analgesic [10], androgen-suppressing [11], anticancer [12, 13], antibacterial, anticonvulsant, antifungal [14], antioxidant [13], anti-inflammatory [10, 15], antiosteoprotic [16], antiulcerogenic, antistress [17], hepatoprotective [18], and sedative activities [19]. However, the investigation about the chemical constituents of *W. chinensis* is not sufficient compared to the other plants in the genus *Wedelia*.

As part of an ongoing search for new biologically active natural products from Vietnamese medicinal plants, we found that a MeOH extract of *W. chinensis* leaves showed significant *in vitro α*-amylase and *α*-glucosidase inhibitory activities. Previously, there have been no reports on either the extracts or isolated components from this species against *α*-amylase and *α*-glucosidase activities. Reported herein are the isolation and structural elucidation of a new megastigmane sesquiterpenoid sulfonic acid (**1**), a new cyclohexylethanoid derivative (**2**), and 7 known compounds (**3–9**) as well as the evaluation of *α*-amylase and *α*-glucosidase activities.

## 2. Materials and Methods

### 2.1. General Experimental Procedures

Optical rotations were measured using a JASCO P-2000 polarimeter (JASCO, Oklahoma, OK, US). IR spectra were obtained on a Bruker TENSOR 37 FT-IR spectrometer (Bruker, Billerica, MA, USA). The ^1^H and ^13^C, HMQC, HMBC, NOESY/ROESY, and COSY NMR spectra were recorded on a 500 MHz Bruker DRX spectrometer (Bruker, Tupper Hall, CA, USA), and the chemical shift (*δ*) was expressed in ppm with reference to the TMS signals. The HRESIMS were obtained using an Agilent 6550 iFunnel Q-TOF LC/MS system (Emeryville, CA, USA). Medium-pressure liquid chromatography (MPLC) was carried out on a Biotage-Isolera  One  system. Column chromatography (CC) was conducted using 65–250 or 230–400 mesh silica gel (Sorbent Technologies, Atlanta, GA, USA), porous polymer gel (Diaion® HP-20, 20–60 mesh, Mitsubishi Chemical, Tokyo, Japan), Sephadex™ LH-20 (Supelco, Bellefonte, PA, USA), octadecyl silica (ODS, 50 *μ*m, Cosmosil 140 C_18_-OPN, Nacalai Tesque), and YMC RP-C_18_ resins (30–50 *μ*m, Fuji Silysia Chemical). Analytical thin-layer chromatography (TLC) systems were performed on precoated silica gel 60 F_254_ plates (1.05554.0001, Merck) and RP-18 F_254S_ plates (1.15685.0001, Merck), and the isolated compounds were visualized by spraying with 10% H_2_SO_4_ in water and then heating for 1.5–2 minutes. All procedures were carried out with solvents purchased from commercial sources, which were used without further purification.

### 2.2. Plant Material

The leaves of *W. chinensis* (Osbeck.) Merr. were collected from Ba Dinh, Ha Noi, Vietnam, in April 2017 and taxonomically identified by Professor Tran Huy Thai (Institute of Ecology and Biological Resources). A voucher specimen (NCCB-2016.55.01) was deposited at the Herbarium of Institute of Marine Biochemistry and Institute of Ecology and Biological Resources, Vietnam Academy of Science and Technology.

### 2.3. Compounds

From the methanolic extract of *W. chinensis*, 9 compounds (**1–9**) were isolated and structurally elucidated. Stock solutions of tested compounds in DMSO were prepared kept at −20°C and diluted to the final concentration in fresh media before each experiment. To not affect the cell growth, the final DMSO concentration did not exceed 0.5% in all experiments.

### 2.4. Extraction and Isolation

The dried leaves of *W. chinensis* (4.4 kg) were cut into pieces and extracted with 95% aqueous MeOH (3 × 6.5 L) under ultrasonic agitation at 90 Hz. The methanol solution was removed of solvent under a vacuum and was filtered through a Büchner funnel to produce a dried brown extract (160 g, A). Since the MeOH extract significantly reduced *α*-amylase and *α*-glucosidase inhibitory activities, it was suspended in distilled water and partitioned between EtOAc (1 L × 3) to obtain EtOAc (16.9 g, B) and aqueous soluble fractions (W).

The EtOAc fraction was separated on silica gel MPLC (column: Biotage® SNAP Cartridge, KP-SIL, 340 g) using the mobile phase of *n*-hexane-acetone (gradient 30 : 70, 50 : 50, 70 : 30, 0 : 100, 15 mL/min, 90 min) to give six fractions (B-1 to B-6). This MPLC procedure was repeated 5 times using the same conditions before further isolation. Fraction B-3 was chromatographed by Sephadex® LH-20 CC (*Φ*25 mm, L 1250 mm) eluted with acetone-H_2_O (gradient 95 : 5, 70 : 30, 50 : 50, v/v) to give three subfractions (B-3.1 to B-3.3) and further purified by YMC RP-C_18_ CC (*Φ*15 mm, L 700 mm) using acetone-H_2_O (1 : 2) as the eluent to pomonic acid (**8**, crystalline powder, 11.2 mg) and pomolic acid (**9**, crystalline powder, 15.4 mg). Next, fraction B‒6 was chromatographed over a silica gel CC (*Φ*12 mm, L 600 mm) eluted with *n*-hexane-EtOAc (2 : 1) to obtain jaceosidin (**7**, pale yellow crystals, 56.8 mg).

The H_2_O fraction was separated using a Diaion HP-20 column (*Φ*100 mm, L 500 mm) and was eluted with a gradient solvent mixture of MeOH-H_2_O (gradient 25 : 75, 50 : 50, 65 : 35, 75 : 25, to pure MeOH, stepwise) to yield five fractions (W-1 to W-5), based on TLC analysis. The fractionation W-4 was separated via silica gel CC (*Φ*30 mm, L 750 mm) and eluted repeatedly with CH_2_Cl_2_-MeOH (0 → 100%) to yield five subfractions (W-4.1 to W-4.5). Subfraction W-4.1 was subjected to a silica gel CC (*Φ*20 mm, L 800 mm with a solvent mixture of CH_2_Cl_2_-MeOH, 9 : 1) and passed over a Sephadex LH-20 column (*Φ*15 mm, L 950 mm) and then through an open YMC RP-C_18_ silica gel column (*Φ*15 mm, L 800 mm, 65 → 100%, H_2_O-MeOH) to afford wednenol (**2**, colorless oil, 12.5 mg), cleroindicin E (**3**, colorless oil, 15.2 mg), and rengyol (**4**, white solid, 46.6 mg). Finally, when the same steps were repeated as above, wednenic (**1**, white powder, 10.6 mg), cornoside (**5**, brown oil, 28.7 mg), and benzyl *β*-D-glucopyranoside-2-sulfate (**6**, amorphous white powder, 86.1 mg) were also obtained by purifying subfraction W-4.5 on YMC RP-C_18_ silica gel (*Φ*20 mm, L 700 mm) and followed by passing over a Sephadex LH-20 column (*Φ*15 mm, L 900 mm) using mixtures of MeOH-H_2_O (1 : 5).

### 2.5. Physical and Spectroscopic Data of Compounds

*Wednenic* (**1**): white powder; [*α*]_*D*_^24^−26.5 (*c* 0.25, MeOH); IR *ν*_max_ (KBr) 3395, 2968, 2930, 1648, 1580, 1511, 1372, 1226, 1163, 1075, and 1040 cm^−1^; HRESIMS (positive-ion mode) *m/z* 345.0987 [M + H]^+^ (cald. for C_13_H_23_O_7_S, 345.0984) and 367.0801 [M + Na]^+^ (cald. for C_13_H_22_NaO_7_S, 367.0803) and (negative-ion mode) *m/z* 225.1482 [M − SO_4_Na]^−^ (cald. for C_13_H_21_O_3_, 225.1490); for ^1^H NMR (CD_3_OD, 500 MHz) and ^13^C NMR (CD_3_OD, 125 MHz) spectroscopic data, see Table 1.

*Wednenol* (**2**): colorless oil; [*α*]_*D*_^24^−47.2 (*c* 0.22, MeOH); IR *ν*_max_ (KBr) 3341, 2950, 1455, 1270, 1168, and 920 cm^−1^; HRESIMS (positive-ion mode) *m/z* 185.1170 [M + H]^+^ (cald. for C_10_H_17_O_3_, 185.1177); for ^1^H NMR (CD_3_OD, 500 MHz) and ^13^C NMR (CD_3_OD, 125 MHz) spectroscopic data, see Table 1.

*Benzyl β-D-glucopyranoside-2-sulfate* (**6**): amorphous white powder; [*α*]_*D*_^24^ 60.8 (*c* 0,25, MeOH); IR *ν*_max_ (KBr): 3595, 3100, 2952, 2850, 1647, 1575, 1511, 1362, 1228, 1153, 1055, and 1010 cm^−1^; ESIMS (negative-ion mode) *m/z* 349,1 [M − H]^−^; ^1^H NMR (500 MHz, CD_3_OD): *δ*_H_ 3.31 (1 H, m, H-5′), 3.44 (1 H, t, *J* = 9.0 Hz, H-3′), 3.66 (1 H, t, *J* = 9.0 Hz, H-4′), 3.71 (1 H, d, *J* = 1.5, 12.0 Hz, H-6′b), 3.91 (1 H, d, *J* = 1.5, 12.0 Hz, H-6′a), 4.17 (1 H, t, *J* = 8.0 Hz, H-2′), 4.53 (1 H, d, *J* = 7.5 Hz, H-1′), 4.74 (1 H, d, *J* = 12.0 Hz, H-7b), 4.93 (1 H, d, *J* = 12.0 Hz, H-7a), 7.25 (1 H, d, *J* = 7.5 Hz, H-4), 7.33 (2 H, d, *J* = 7.5 Hz, H-3 and H-5), and 7.47 (2 H, br t, *J* = 7.5 Hz, H-2 and H-6); ^13^C NMR (125 MHz, CD_3_OD): *δ*_C_ 138.9 (C-1), 128.8 (C-2 and C-6), 129.1 (C-3 and C-5), 128.4 (C-4), and 71.5 (C-7); *Glc*: 101.0 (C-1′), 81.4 (C-2′), 77.4 (C-3′), 71.5 (C-4′), 77.6 (C-5′), and 62.6 (C-6′).

### 2.6. Preparation of (*S*)- and (*R*)-MTPA Ester Derivatives of **1**

Compound **1** (3.0 mg) was dissolved in 2.5 mL of anhydrous CH_2_Cl_2_. Dimethylaminopyridine (35 mg), triethylamine, and (*R*)-MTPA chloride (30 *μ*L) were then added in sequence. The reaction mixture was stirred for 3 h at room temperature and then quenched by the addition of 1 mL of aqueous MeOH. The solvents were removed under vacuum, and the residue was passed through a small silica gel column using CH_2_Cl_2_-MeOH (100 : 1) as the eluent to provide the (*S*)-MTPA ester of **1** (**1a**, 1.2 mg). The (*R*)-MTPA derivative (**1b**, 1.5 mg) was prepared with (*S*)-MTPA chloride and purified in the same manner.

(*S*)-MTPA ester derivative of **1** (**1a**): 1 H·NMR (CD3OD, 500 MHz): *δ*_H_ 7.613–7.421 (10 H, overlap, aromatic protons), 3.630 (3 H, s, OCH3), 1.501 (1H, dd, *J* = 3.5, 12.5 Hz, H-2a), 1.859 (1 H, t, *J* = 12.5 Hz, H-2b), 4.693 (1 H, dt, *J* = 3.5, 12.5 Hz, H-3), 5.767 (1 H, d, *J* = 3.5 Hz, H-4), 6.418 (1 H, dd, *J* = 1.0, 16.5 Hz, H-7), 5.901 (1 H, dd, *J* = 6.0, 16.5 Hz, H-8), 5.983 (1 H, dd, *J* = 6.0, 12.5 Hz, H-9), 1.431 (3 H, d, *J* = 6.0 Hz, H-10), 1.202 (3 H, s, H-11), 1.050 (3 H, s, H-12), and 1.315 (3 H, s, H-13).

(*R*)-MTPA ester derivative of **1** (**1b**): 1 H·NMR (CD3OD, 500 MHz): *δ*_H_ 7.613–7.401 (10 H, overlap, aromatic protons), 3.58 (3 H, s, OCH3), 1.516 (1 H, dd, *J* = 3.5, 12.5 Hz, H-2a), 1.878 (1 H, t, *J* = 12.5 Hz, H-2b), 4.676 (1 H, dt, *J* = 3.5, 12.5 Hz, H-3), 5.767 (1 H, d, *J* = 3.5 Hz, H-4), 6.395 (1 H, dd, *J* = 1.0, 16.5 Hz, H-7), 5.846 (1 H, dd, *J* = 6.0, 16.5 Hz, H-8), 5.982 (1 H, dd, *J* = 6.0, 12.5 Hz, H-9), 1.463 (3 H, d, *J* = 6.0 Hz, H-10), 1.174 (3 H, s, H-11), 1.014 (3 H, s, H-12), and 1.328 (3 H, s, H-13).

### 2.7. Assay for *α*-Amylase Inhibition

The porcine pancreas *α*-amylase (A3176, Sigma-Aldrich) enzyme inhibitory activity was carried out according to the standard method with minor modifications [20–22]. The substrate was prepared by boiling 100 mg potato starch in 5 mL phosphate buffer (pH 7.0) for 5 min and then cooling to room temperature. The sample (2 mL dissolved in DMSO) and substrate (50 mL) were mixed in 30 mL of 0.1 M phosphate buffer (pH 7.0). After 5 min preincubation, 5 mg/mL *α*-amylase solution (20 mL) was added, and the solution was incubated at 37°C for 15 min. The reaction was stopped by adding 50 mL of 1 M·HCl, and then, 50 mL of iodine solution was added. The absorbances were measured at 650 nm by a microplate reader. Acarbose was used as a positive control. The inhibitory activity was calculated by the following equation: *α*-amylase inhibitory activity (%) = (1–A/A0) × 100, where A is the absorbance of the sample and A0 is the absorbance of the blank, respectively. The IC_50_ value was calculated by GraphPad Prism.

### 2.8. Assay for *α*-Glucosidase Inhibition

The yeast *α*-glucosidase (G0660, Sigma-Aldrich) inhibition assay was performed using the substrate *p*-nitrophenyl-*α*-D-glucopyranoside according to the previously described method [21–23]. Briefly, samples and acarbose were prepared by dissolving at 2 mg/mL (with extracts) or 0.8 mM (with pure compound) in DMSO, and 0.5 U/mL *α*-glucosidase (40 mL) was mixed in 120 mL of 0.1 M phosphate buffer (pH 6.8). After 5 min preincubation, 5 mM *p*-nitrophenyl-*α*-D-glucopyranoside solution (40 mL) was added, and the solution was incubated at 37°C for 30 min. Pipette the following reagents into a 96-well plate. Each concentration of samples was carried out in triplicate. The absorbance of released 4-nitrophenol was measured at 405 nm by using a microplate reader (xMark, Bio-Rad, USA). Acarbose was used as the positive control. The inhibitory activity was calculated by the following equation: *α*-glucosidase inhibitory activity (%) = (1–A/A0) × 100, where A is the absorbance of the sample and A0 is the absorbance of the blank, respectively. The IC_50_ value was calculated by GraphPad Prism.

### 2.9. Data Expression and Statistical Analysis

Data were expressed as mean value ± standard deviation (SD) of blood glucose. Data were evaluated using two-way ANOVA followed by Dunnett's multiple comparison test, and groups were considered significantly different if *P* < 0.05. All data are presented as mean ± SD.

## 3. Results and Discussion

### 3.1. Identification of Compounds **1–9**

**Figure 1:**
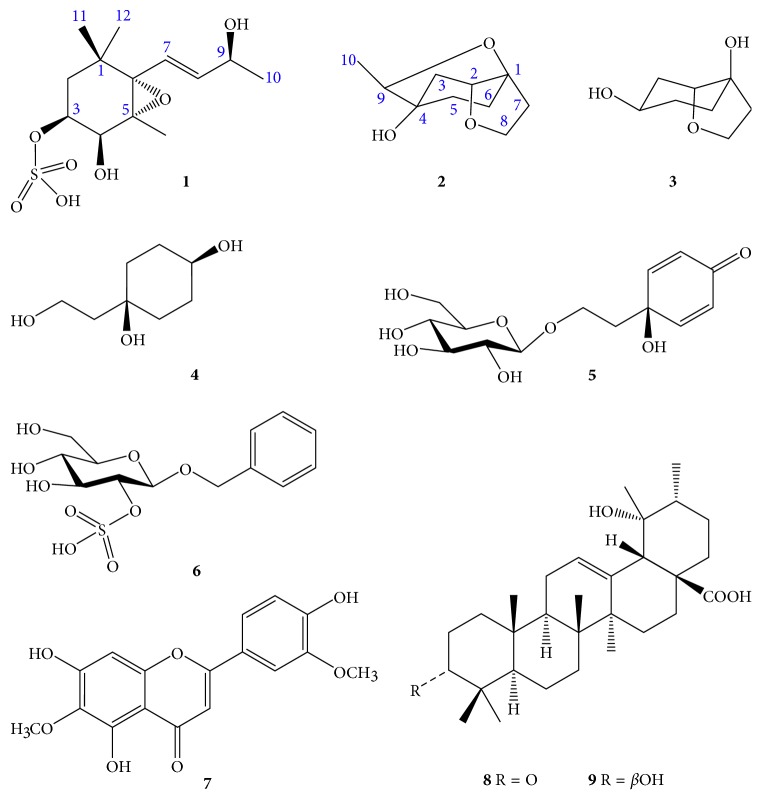
Structures of compounds **1–9** isolated from *W. chinensis*.

A MeOH extract from the leaves of *W. chinensis* was suspended in H_2_O and fractionated successively with EtOAc-soluble fraction, and then, each fraction was evaluated for *α*-amylase and *α*-glucosidase activities. The EtOAc-soluble fraction and water layer were chosen for subsequent studies, which resulted in the isolation of two new compounds (**1–2**), together with seven known compounds (**3–9**; Figure 1). Moreover, compounds **1–6** were reported for the first time from this species and from the genus *Wedelia*.

Wednenic (**1**) was obtained as a white powder with a negative optical rotation ([α]_D_^24^−26.5, *c* 0.25, MeOH), and the molecular formula, C_13_H_22_O_7_S, was determined by HRESIMS, with a protonated molecular ion peak at *m/z* 345.0987 [M + H]^+^ and a sodium adduct molecular ion peak at *m/z* 367.0801 [M + Na]^+^. The fragment ion peak at *m/z* 225.1482 [M − SO_4_Na]^−^ in the (−)HRESIMS spectrum showed the presence of a sulfate group in **1**. A detailed assessment of the NMR data indicated that **1** is a megastigmane, a compound class known as components of plant species.

The ^1^H NMR spectrum (Table 1) of **1** exhibited signals for four methyl groups [*δ*_H_ 1.24 (d, *J* = 6.0 Hz, H-10), 1.03 (s, H-11), 1.13 (s, H-12), and 1.28 (s, H-13)], a pair of methylene protons [*δ*_H_ 1.47 (dd, *J* = 3.5, 12.5 Hz, H-2a) and 1.84 (t, *J* = 12.5 Hz, H-2b)], three oxygenated methines [*δ*_H_ 4.60 (ddd, *J* = 3.0, 3.5, 12.5 Hz, H-3), 4.27 (dd, *J* = 1.0, 3.0 Hz, H-4), and 4.31 (dd, *J* = 6.0, 12.5 Hz, H-9)], and two olefinic protons [*δ*_H_ 5.92 (dd, *J* = 1.0, 16.5 Hz, H-7) and 5.69 (dd, *J* = 6.0, 16.5 Hz, H-8)]. The larger coupling constant (*J* = 16.5 Hz) between H-7 and H-8 indicates that the geometry of the Δ^7^ double bond is *E*. The ^13^C NMR and DEPT spectra (Table 1) showed 13 carbon signals of four methyls [*δ*_C_ 23.7 (C-10), 24.8 (C-11), 29.5 (C-12), and 17.1 (C-13)], a methylene [(*δ*_C_ 37.9 (C-2)], an oxygenated methine bearing a sulfate group [*δ*_C_ 75.5 (C-3)], two oxygenated methines [*δ*_C_ 71.6 (C-4) and 68.5 (C-9)], and three nonprotonated carbons [*δ*_C_ 35.5 (C-1), 69.4 (C-5), and 71.3 (C-6)], together with a pair of *trans*-olefinic methine carbons [*δ*_C_ 125.6 (C-7) and 139.3 (C-8)]. Based on these data, a megastigmane sesquiterpenoid sulfonic acid has been determined for **1**.

**Figure 2:**
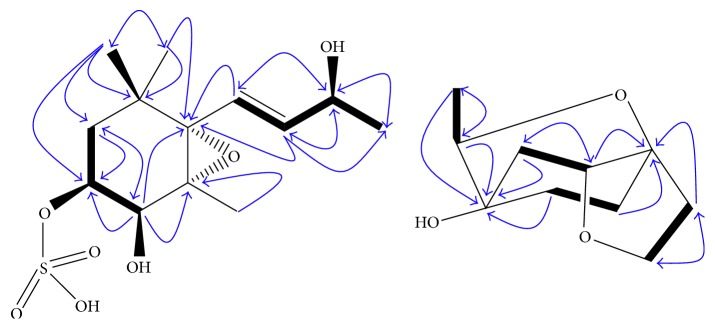
Key HMBC (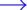) and COSY (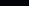) correlations of **1** and **2**.

Interpretation of the COSY and HSQC spectra of **1** revealed the presence of two partial structures, “C-2/C-3/C4” and “C-7/C-8/C-9/C-10” (Figure 2). These two partial structures were connected through a nonprotonated carbon (C-6) on the basis of HMBC correlations of H-4, H-7, H-8, H-12, and H-13 to C-6. The HMBC correlations from H_3_-11 and H_3_-12 to C-1, C-2, and C-6 indicated that C-2, C-11, C-12, and C-6 were all connected with C-1. The chemical shifts and coupling constants of **1** were in good agreement with those of (3*S*,4*S*,5*R*,6*S*,9*S*,7*E*)-megastigman-7ene-5,6-epoxy-3,4,9-triol 9-O-*β*-D-glucopyranoside recorded in the same deuterated solvent [24] but quite different from the data of the C-4. The substitution at C-3 and C-4 was tentatively identified as a sulfonate group and a hydroxyl group based on the HMBC correlations, which supported the total structure of **1** (Figure 2).

**Figure 3:**
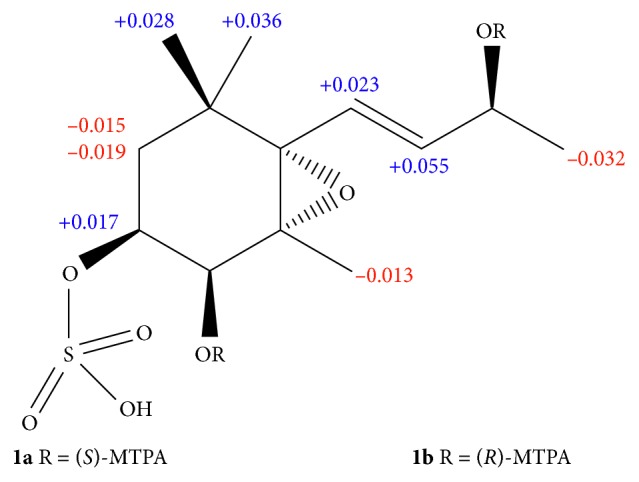
Δ*δ*_H (*S*−*R*)_ values (in ppm) for MTPA esters of **1**.

The relative configuration of **1** was deduced from analysis of coupling constants and the NOESY spectrum (except for C-9), which were both consistent with a chair conformation for the cyclohexane ring. The *α*-orientation of H-3 was deduced from the cross-peak of H-3 to H-2a/H_3_-12 and H-7 and H-2b to H-11 and H-8 in the NOESY spectrum, and the larger coupling constant *J* = 12.5 Hz of H-3 (*δ*_H_ 4.60) with one of the H-2a protons (*δ*_H_ 1.84) indicated that H-3 was axial. Moreover, the NOESY correlation between H-4 and H-3 indicated that H-4 and H-3 were axial bonds, which established its *α*-orientation. Additionally, the coupling constants at H-3 [*δ*_H_ 4.60 (1 H, ddd, *J* = 3.0, 3.5, 12.5 Hz)] and H-4 [*δ*_H_ 4.27 (1 H, dd, *J* = 1.0, 3.0 Hz)] of **1** were in good agreement with those of (3*S*,4*S*,5*R*,6*S*,9*S*,7*E*)-megastigman-7ene-5,6-epoxy-3,4,9-triol 9-O-*β*-D-glucopyranoside [*δ*_H_ 3.79 (1 H, ddd, *J* = 3.0, 3.0, 12.0 Hz, H-3) and 3.88 (1 H, dd, *J* = 1.0, 3.0 Hz, H-4)] recorded in the same deuterated solvent. These findings confirmed that two compounds have the same configurations at C-3 and C-4 [24]. The chemical shifts of C-5 (*δ*_C_ 69.4) and C-6 (*δ*_C_ 71.3) corresponded well to the similar signals observed in the ^13^C NMR spectra (in CD_3_OD) with (3*S*,4*S*,5*R*,6*S*,9*S*,7*E*)-megastigman-7ene-5,6-epoxy-3,4,9-triol 9-O-*β*-D-glucopyranoside [*δ*_C_ 69.7 (C-5) and 71.6 (C-6)] [24] and the major difference from the ^13^C NMR spectrum (in CD_3_OD) with 5*S*,6*R* configurations of (3*S*,4*S*,5*S*,6*R*,7*E*,9*S*)-5,6-epoxy-3,4,9-trihydroxy-7-megastigmen-3-O-*β*-D-glucopyranoside (komaroveside C) [*δ*_C_ 68.2 (C-5) and 70.4 (C-6)] [25]. These data clearly indicated the presence of 5*R*,6*S* configurations in **1**. To determine the absolute configuration of C-9 in **1**, (*S*)- and (*R*)-MTPA esters (**1a** and **1b**) were prepared. Significant Δ*δ* values (Δ*δ* = *δ*_*S*-MTPA-ester_ − *δ*_*R*-MTPA-ester_) were observed for the proton signal adjacent to C-9, as shown in Figure 3. According to the rule of the modified Mosher's method [26, 27], the absolute configuration at C-9 in **1** was assigned *S*-form. On the basis of the abovementioned data, the structure of **1** was elucidated to be (3*S*,4*R*,5*R*,6*S*,9*S*,7*E*)-megastigman-5,6-epoxy-7-ene,4,9-diol,3-sulfonic acid.

A molecular formula of C_10_H_16_O_3_ was established for wednenol (**2**) based on the presence of 10 signals in its ^13^C NMR spectrum and the HRESIMS protonated molecular ion peak at *m/z* 185.1170 [M + H]^+^ (cald. 185.1178). In the ^1^H NMR spectrum, signals were observed for a secondary methyl group [*δ*_H_ 1.15 (d, *J* = 6.5 Hz, H-10)], two oxygen-bearing methine groups [*δ*_H_ 4.00 (dd, *J* = 6.0, 9.5 Hz, H-2) and 3.51 (q, *J* = 6.5 Hz, H-9)], oxygenated methylene groups [*δ*_H_ 3.95 (ddd, *J* = 3.0, 8.0, 9.5 Hz, H-8a) and 4.05 (dd, *J* = 8.0, 8.5 Hz, H-8b)], and four methylene groups (Table 1). Its ^13^C NMR spectrum exhibited ten carbon signals, including a methyl [*δ*_C_ 17.0 (C-10)], two methines [*δ*_C_ 83.3 (C-2) and 74.3 (C-9)], an oxygenated methylene [*δ*_C_ 66.4 (C-8)], and four methylenes [*δ*_C_ 37.5 (C-3), 29.2 (C-5), 30.5 (C-6), and 36.0 (C-7)], suggesting that **2** was a cyclohexylethanoid derivative [28].

Analysis of the ^1^H and ^13^C NMR data of **2** (Table 1) suggested that this compound shares several structural similarities with cleroindicin E (**3**) [28], except for major differences in the resonances associated with the additional methyl and oxygenated methine groups. The ^1^H-^1^H COSY spectrum of **2** was observed to exhibit proton correlations between H-5 and H-6, between H-7 and H-8, between H-2 and H-3, and between H-9 and H-10 (Figure 2). In the HMBC spectrum, cross-peaks between H-10 (*δ*_H_ 1.15) and C-4 (*δ*_C_ 75.4), and H-10 (*δ*_H_ 1.15) and C-9 (*δ*_C_ 74.3), between H-9 (*δ*_H_ 3.51) and C-3 (*δ*_C_ 37.5), C-4 (*δ*_C_ 75.4), and C-5 (*δ*_C_ 29.2) clearly indicated the positions of the methyl and oxygenated methine groups. Moreover, the C-1 resonance was shifted downfield, from *δ*_C_ 75.8 in **3** to *δ*_C_ 79.4 in **2**. The downfield shift of C-1 may be explained by the presence of a C-1‒C-9 ether linkage in **2**. These findings suggested that one cyclohexylethanoid unit in **2** should have a 1,2,4-trioxygenated-cyclohexylethanoid structure. Detailed analysis of the other COSY and HSQC spectra unambiguously identified the planar structure of **2** (Figure 2). In the NOESY spectrum of **2**, the doublet of doublets belonging to H-2 [*δ*_H_ 4.00 (1H dd, *J* = 6.0, 9.5 Hz)] was observed, which correlated with H-3a and H-7a, and the coupling constant *J* = 6.0 Hz of H-2 (*δ*_H_ 4.00) with one of the H-3a protons (*δ*_H_ 1.39) indicated that H-2 was equatorial orientation. Additionally, the NOESY correlation of H-3a and H-9 and their coupling constant (*J* = 6.5 Hz) suggested that H-9 was also an equatorial bond. Thus, the structure of wednenol was elucidated as **2**.

On comparison of their physical and spectroscopic data with published values, the known compounds were identified as cleroindicin E (**3**) [29], rengyol (**4**) [30], cornoside (**5**) [28], benzyl *β*-D-glucopyranoside-2-sulfate (**6**) [31], jaceosidin (**7**) [32], pomonic acid (**8**) [33], and pomolic acid (**9**) [34].

### 3.2. *α*-Amylase and *α*-Glucosidase Inhibitory Activities of Compounds **1–9**

The inhibitory effects of the isolated compounds against porcine pancreas *α*-amylase and yeast *α*-glucosidase were evaluated in comparison with the antidiabetic acarbose. *α*-Glucosidase is the key catalyzing enzyme involved in the process of carbohydrate digestion and glucose release. Inhibition of *α*-glucosidase is one very effective way of delaying glucose absorption and lowering the postprandial blood glucose level, which can potentially suppress the progression of DM.

**Figure 4:**
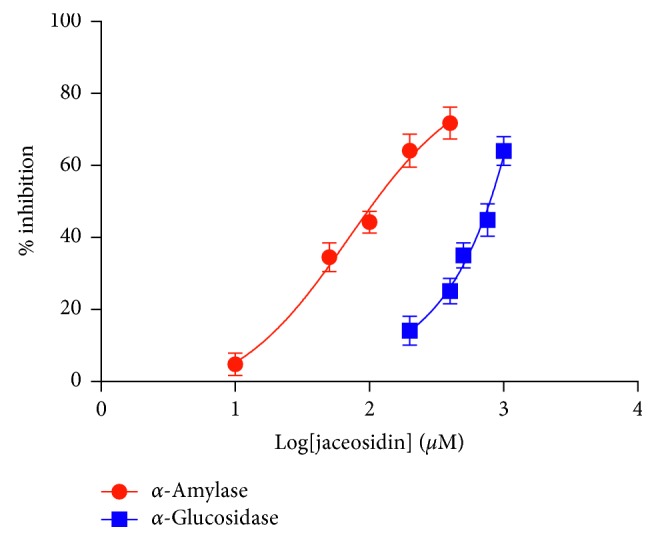
IC_50S_ of **7** against *α*-amylase and *α*-glucosidase. Activity data points (absorbance) were plotted as mean ± SD (*n*=3).

The isolated flavonoid (**7**) showed the most active *α*-amylase and *α*-glucosidase inhibitory activities with IC_50_ values of 112.8 ± 15.1 and 785.9 ± 12.7 *μ*g/mL, respectively (Table 2 and Figure 4). This is in agreement with a recent report of the *α*-amylase and *α*-glucosidase inhibitory activities in other flavonoids [35–37]. Compounds **1**, **8**, and **9** showed moderate inhibitory effects against *α*-amylase and *α*-glucosidase, when compared with those of a standard reference drug, acarbose, with IC_50_ values of 124.0 ± 21.3 *μ*g/mL (against *α*-amylase) and 642.6 ± 46.4 *μ*g/mL (against *α*-glucosidase). In contrast, other compounds (**2–6**) showed weak or did not show any effect on both enzymes. These results indicated that the megastigmane, flavonoids, and triterpenoids exhibited high inhibitory activity but were not sufficient to clarify the structure-activity relationships between derivatives. Further research is required to clarify their potential selective *α*-amylase and *α*-glucosidase activities.

## 4. Conclusion

In conclusion, the present work reported for the first time the *α*-amylase and *α*-glucosidase inhibitory effects of *W. chinensis*, in support of their ethnomedicinal use for diabetes. This report partly defines the reason on why these medicinal plants possess antidiabetic properties and could provide a scientific warrant for their application as health supplementary herbal products for diabetes treatment and prevention.

## Figures and Tables

**Table 1:** ^1^H and ^13^C NMR spectroscopic data of **1** and **2** in CD_3_OD.

Position	**1**		**2**	
	*δ*_C_^a^	*δ*_H_^b^ mult. (*J* in Hz)	*δ*_C_^a^	*δ*_H_^b^ mult. (*J* in Hz)
1	35.5, C	—	79.4, C	—
2	37.9, CH_2_	1.47 dd (3.5, 12.5)	83.3, CH	4.00 dd (6.0, 9.5)
		1.84 t (12.5)		
3	75.5, CH	4.60 ddd (3.0, 3.5, 12.5)	37.5, CH_2_	1.39 dd (6.0, 13.5)
				1.80 m
4	71.6, CH	4.27 dd (1.0, 3.0)	75.4, C	—
5	69.4, C	—	29.2, CH_2_	1.53 ddd (4.5, 9.5, 13.5)
				1.66 m
6	71.3, C	—	30.5, CH_2_	1.93 ddd (4.0, 11.5, 13.5)
				2.09 ddd (4.0, 7.5, 13.5)
7	125.6, CH	5.92 dd (1.0, 16.5)	36.0, CH_2_	1.76 ddd (4.0, 8.0, 12.5)
				2.22 ddd (8.0, 9.5, 12.5)
8	139.3, CH	5.69 dd (6.0, 16.5)	66.4, CH_2_	3.95 ddd (3.0, 8.0, 9.5)
				4.05 dd (8.0, 8.5)
9	68.5, CH	4.31 dd (6.0, 12.5)	74.3, CH	3.51 q (6.5)
10	23.7, CH_3_	1.24 d (6.0)	17.0, CH_3_	1.15 d (6.5)
11	24.8, CH_3_	1.03 s	—	—
12	29.5, CH_3_	1.13 s	—	—
13	17.1, CH_3_	1.28 s	—	—

^a^125 MHz; ^b^500 MHz. Assignments were made using the HMQC, HMBC, COSY, and NOESY spectra.

**Table 2:** Inhibitory effects of selected compounds against *α*-amylase and *α*-glucosidase activities (IC_50_ ± SD, *μ*g/mL).

Compounds^a^	*α*-Amylase	*α*-Glucosidase
**1**	436.8 ± 28.6	915.6 ± 36.5
**7**	112.8 ± 15.1	785.9 ± 12.7
**8**	420.7 ± 25.2	—
**9**	395.6 ± 18.3	821.4 ± 55.2
Acarbose^b^	124.0 ± 21.3	642.6 ± 46.4

^a^Compounds were tested in a set of experiments three times. For different versus control group, *P* < 0.05. ^b^Acarbose was used as a positive control. (‒): no inhibition (less than 10% inhibition).

## Data Availability

The data used to support the findings of this study are available from the corresponding author upon request.

## Supplementary Materials

Supplementary Materials HRESIMS, ^1^H, ^13^C, and 2D NMR spectra of new compounds **1–2**; ^1^H and ^13^C NMR data of compounds **3–5** and **7**.
