# Supplementary material for: α-Amylase and α-Glucosidase Inhibitory Activities of Chemical Constituents from Wedelia chinensis (Osbeck.) Merr. Leaves
Source: J Anal Methods Chem. 2018 May 9;2018:2794904. doi: 10.1155/2018/2794904 (PMC5971303; doi:10.1155/2018/2794904)
Supplement: Supplementary Materials — HRESIMS, 1H, 13C, and 2D NMR spectra of new compounds 1–2; 1H and 13C NMR data of compounds 3–5 and 7. [file 2794904.f1.pdf]

# SUPPORTING INFORMATION

## α-Amylase and α-Glucosidase Inhibitory Activities of Chemical Constituents from *Wedelia chinensis* (Osbeck.) Merr. Leaves


Nguyen Phuong Thao,[1,*] Pham Thanh Binh,[1] Nguyen Thi Luyen,[1,2] Ta Manh Hung,[3] Nguyen Hai Dang,[1,2] Nguyen Tien Dat [2,4,*]

[1] *Advanced Center for Bio-organic Chemistry, Institute of Marine Biochemistry (IMBC), Vietnam Academy of Science and Technology (VAST), 18 Hoang Quoc Viet, Caugiay, Hanoi, Vietnam*

[2] *Graduate University of Science and Technology, VAST, 18 Hoang Quoc Viet, Caugiay, Hanoi, Viet Nam*

[3] *National Institute of Drug Quality Control (NIDQC), 48 Hai Ba Trung, Hoankiem, Hanoi, Vietnam*

[4] *Center for Research and Technology Transfer, VAST, 18 Hoang Quoc Viet, Caugiay, Hanoi, Viet Nam*

* Correspondence should be addressed to:

Tel: +84 4 37917053; Fax: +84 4 37917054; *E-mail addresses*: thaonp@imbc.vast.vn (N.P. Thao), ngtiend@imbc.vast.vn (N.T. Dat).

# List of Content

**Cotents** **Pages**


**Figure S1**. HRESIMS spectrum (MeOH) of compound **1**…………………………………... S-3

**Figure S2.** $^{1}$H NMR spectrum ($CD_3OD$, 500 MHz) of compound **1**……………………... S-3

**Figure S3.** $^{13}$C NMR spectrum ($CD_3OD$, 125 MHz) of compound **1**…………………….. S-4

**Figure S4**. $^{13}$C NMR spectrum ($CD_3OD$, 125 MHz) of compound **1**…………………….. S-4

**Figure S5.** HMQC spectrum ($CD_3OD$, 500 MHz) of compound **1**……………………….. S-5

**Figure S6.** HMBC spectrum ($CD_3OD$, 500 MHz) of compound **1**……………………….. S-5

**Figure S7.** COSY spectrum ($CD_3OD$, 500 MHz) of compound **1**………………………... S-6

**Figure S8.** ROESY spectrum ($CD_3OD$, 500 MHz) of compound **1**……………………… S-6

**Figure S9**. HRESIMS spectrum (MeOH) of compound **2**. ………………………………. S-7

**Figure S10.** $^{1}$H NMR spectrum ($CD_3OD$, 500 MHz) of compound **2**……………………. S-7

**Figure S11.** $^{13}$C NMR spectrum ($CD_3OD$, 125 MHz) of compound **2**…………………… S-8

**Figure S12**. DEPT spectrum ($CD_3OD$, 125 MHz) of compound **2**……………………… S-8

**Figure S13.** HMQC spectrum ($CD_3OD$, 500 MHz) of compound **2**……………………… S-9

**Figure S14.** HMBC spectrum ($CD_3OD$, 500 MHz) of compound **2**……………………… S-9

**Figure S15.** COSY spectrum ($CD_3OD$, 500 MHz) of compound **2**………………………. S-10

**Figure S16.** NOESY spectrum ($CD_3OD$, 500 MHz) of compound **2**…………………….. S-10

$^{1}$H and $^{13}$C NMR spectroscopic data of isolated compounds **3–5** and **7–12**……….............. S-11

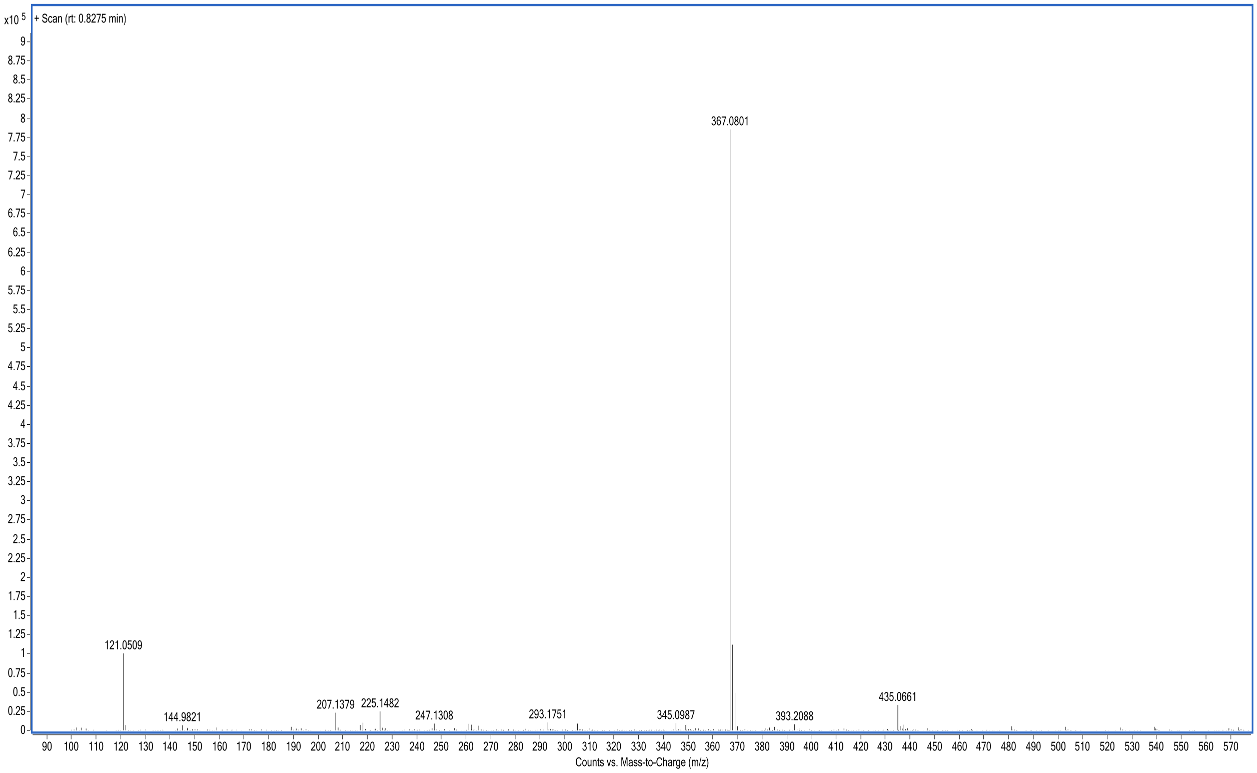


**Figure S1**. HRESIMS spectrum (MeOH) of compound **1**.

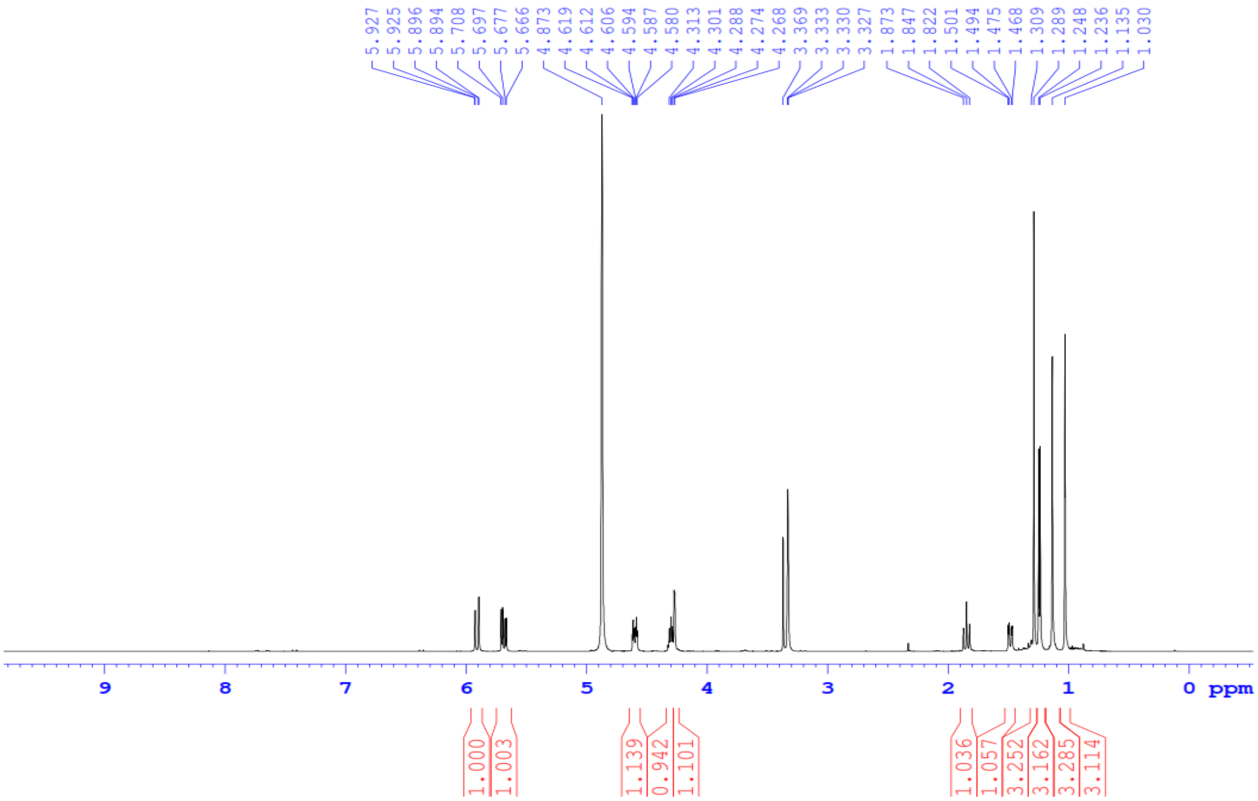


**Figure S2**. $^1$H NMR spectrum ($CD_3OD$, 500 MHz) of compound **1**.

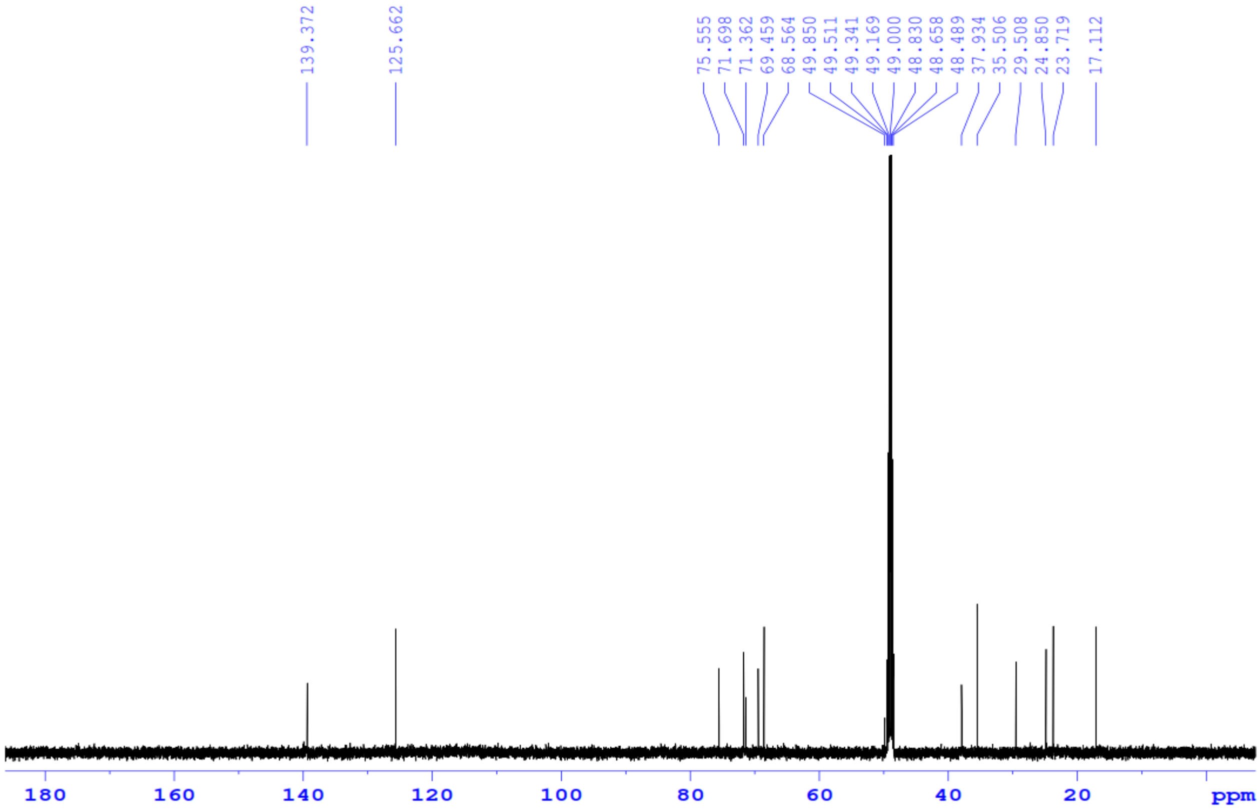


**Figure S3**. $^{13}$C NMR spectrum ($CD_3OD$, 125 MHz) of compound **1**.

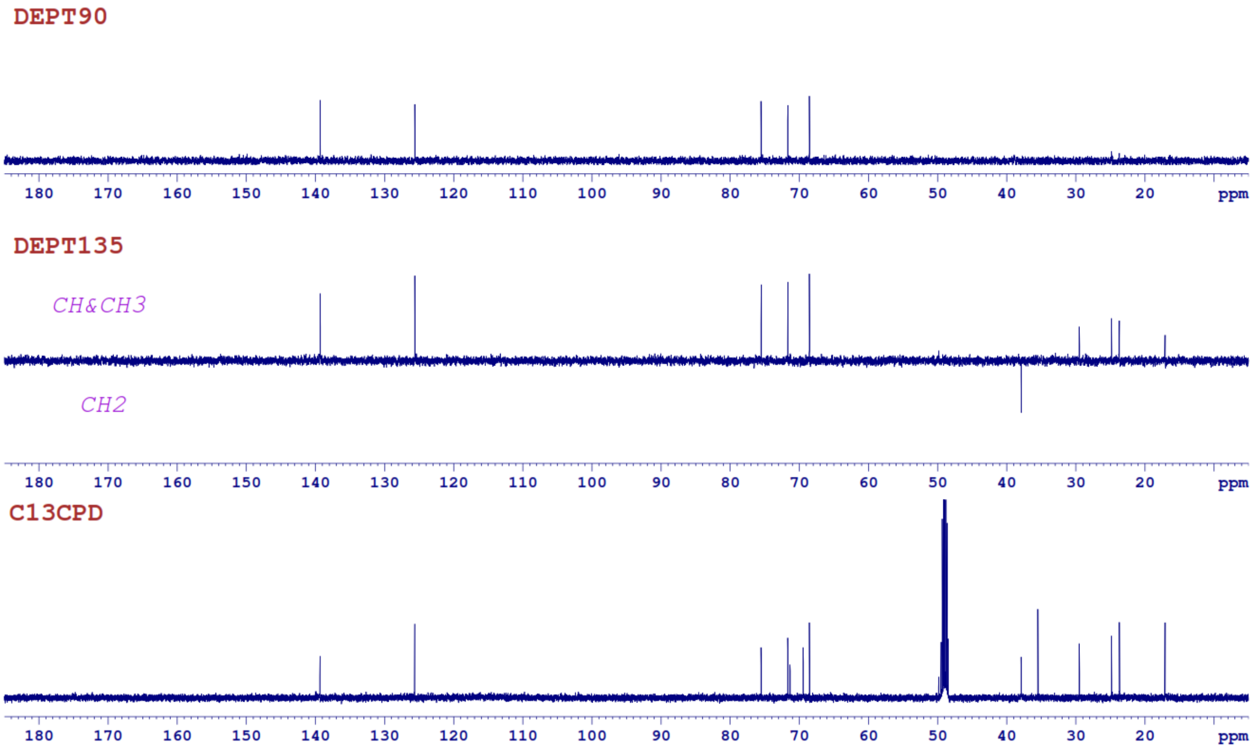


**Figure S4**. DEPT spectrum ($CD_3OD$, 125 MHz) of compound **1**.

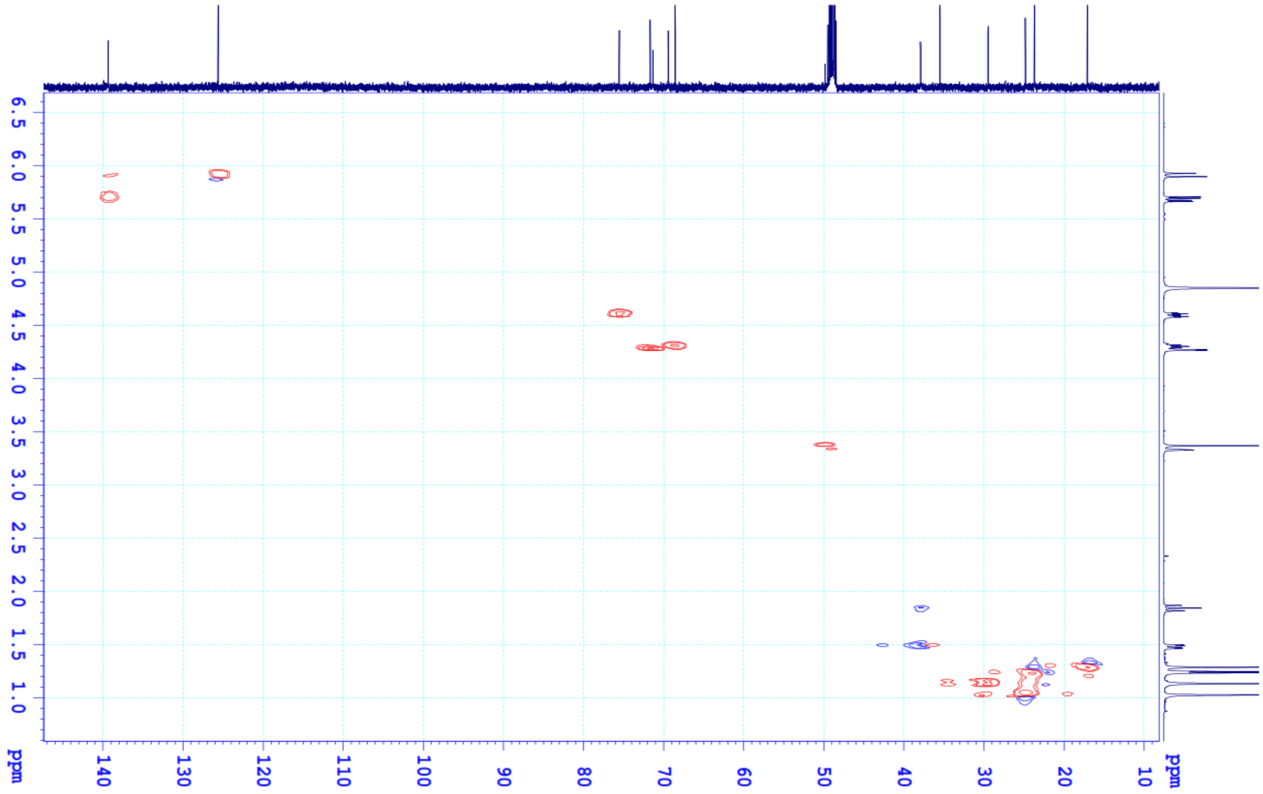

**Figure S5**. HMQC spectrum ($CD_3OD$, 500 MHz) of compound **1**.

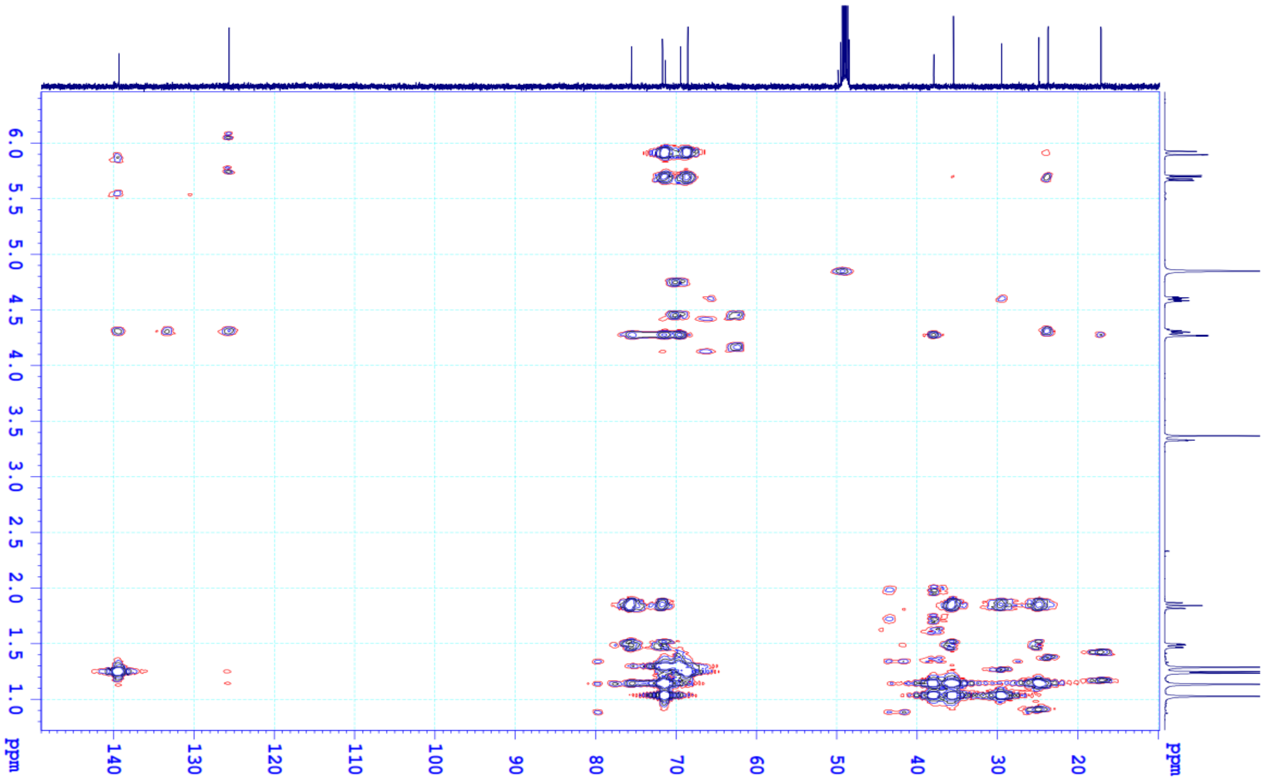

**Figure S6**. HMBC spectrum ($CD_3OD$, 500 MHz) of compound **1**.

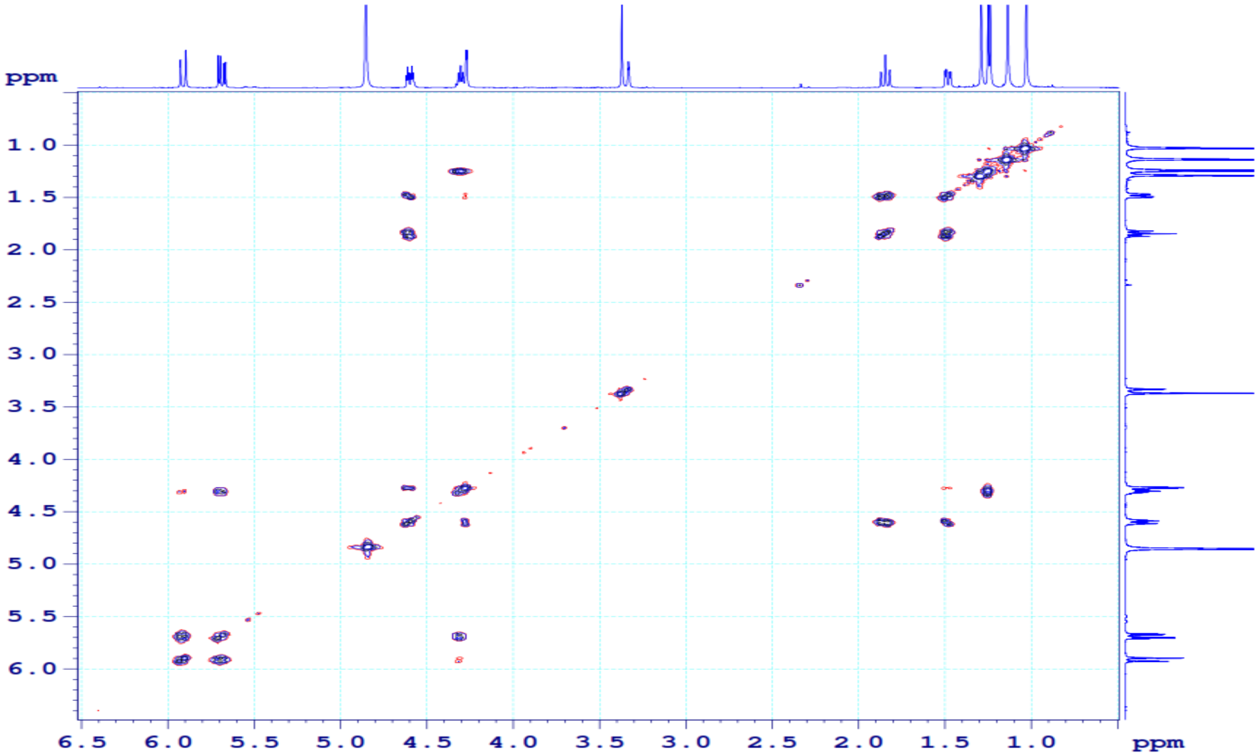


**Figure S7**. COSY spectrum ($CD_3OD$, 500 MHz) of compound **1**.

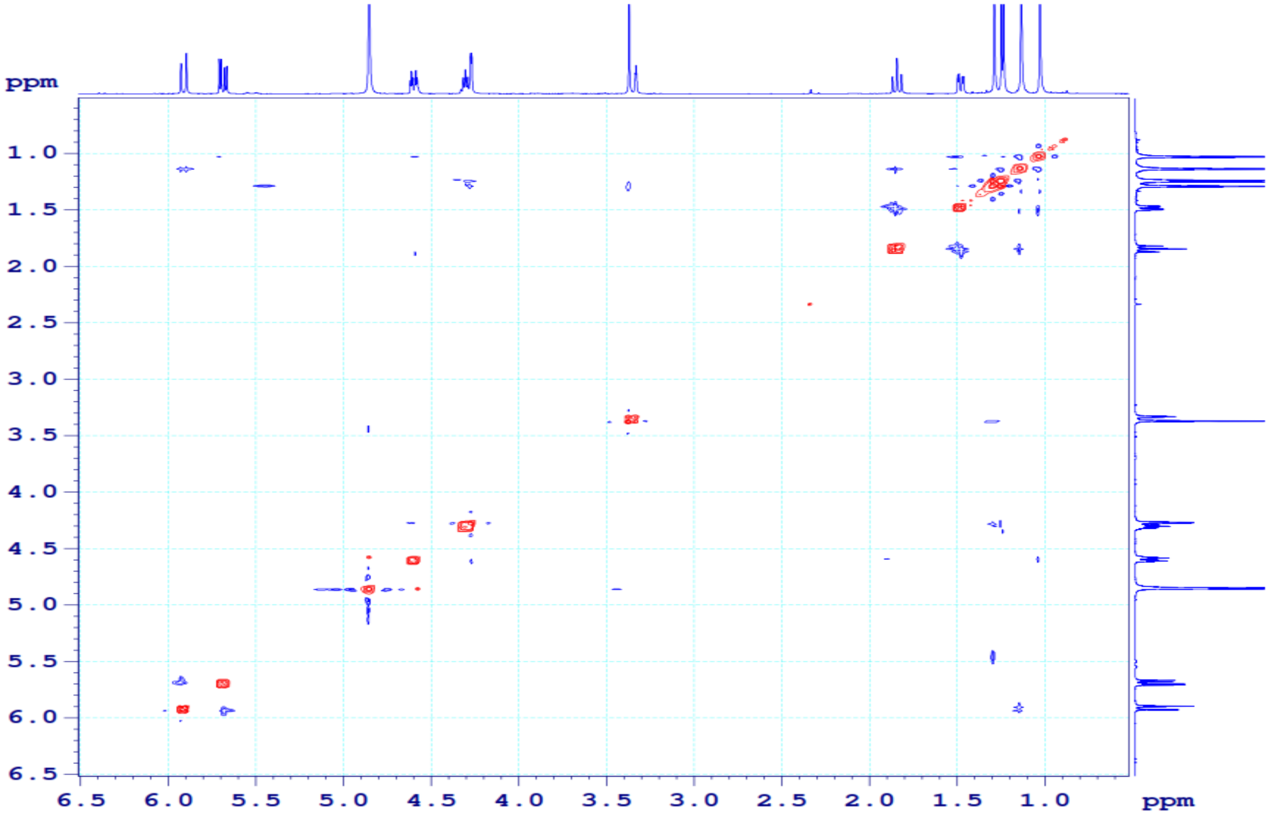


**Figure S8**. NOESY spectrum ($CD_3OD$, 500 MHz) of compound **1**.

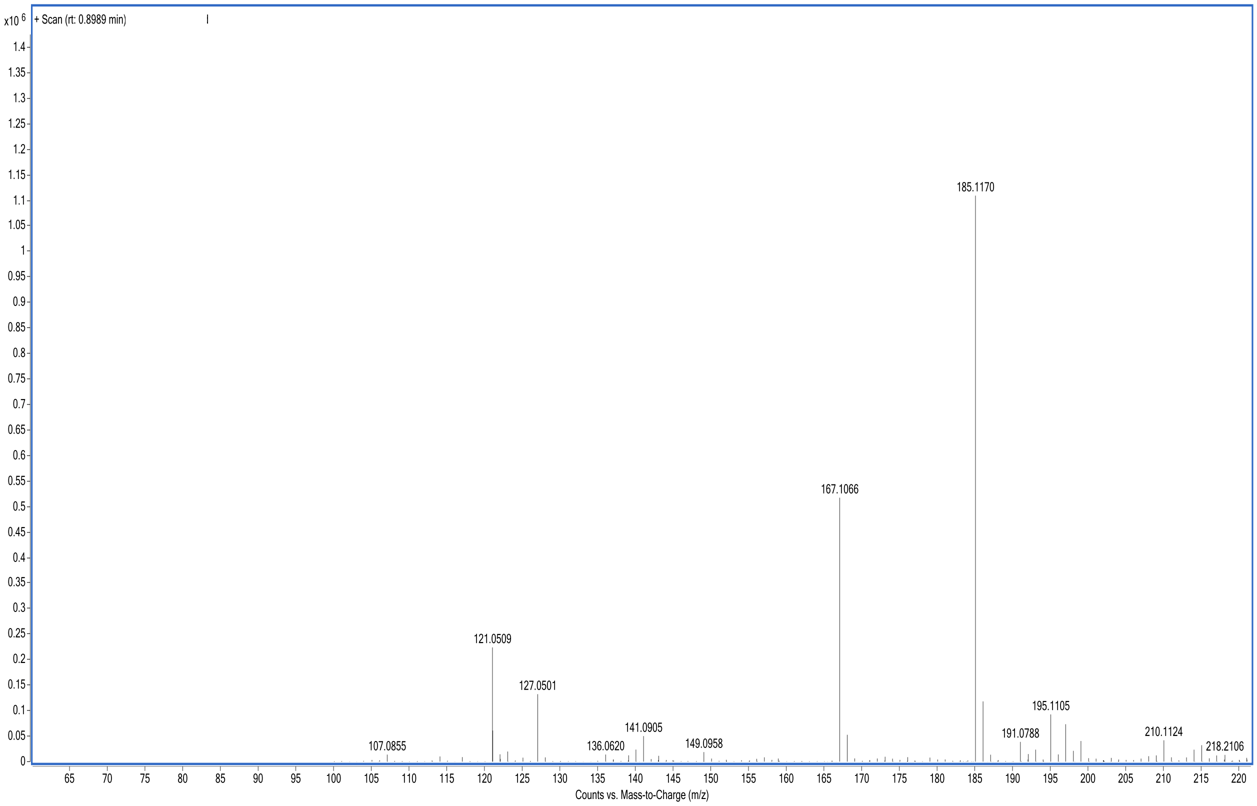


**Figure S9**. HRESIMS spectrum (MeOH) of compound **2**.

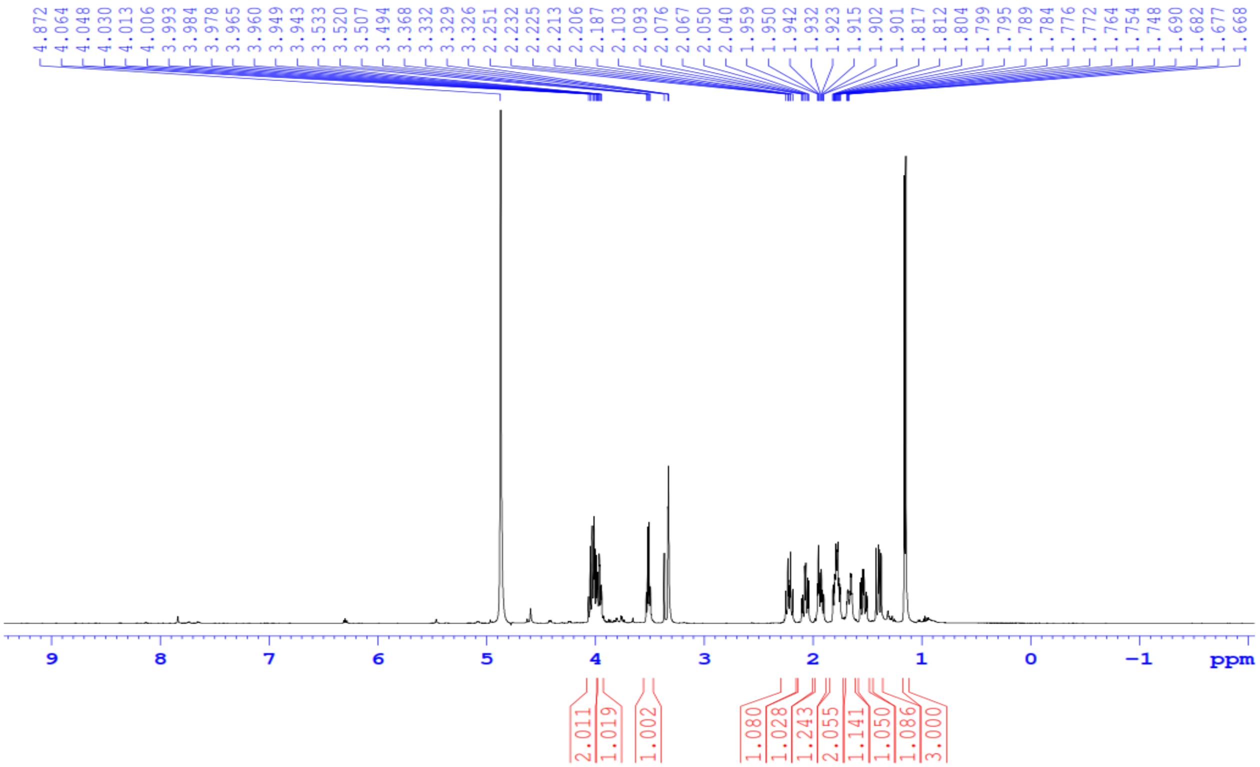


**Figure S10**. $^{1}$H NMR spectrum ($CD_3OD$, 500 MHz) of compound **2**.

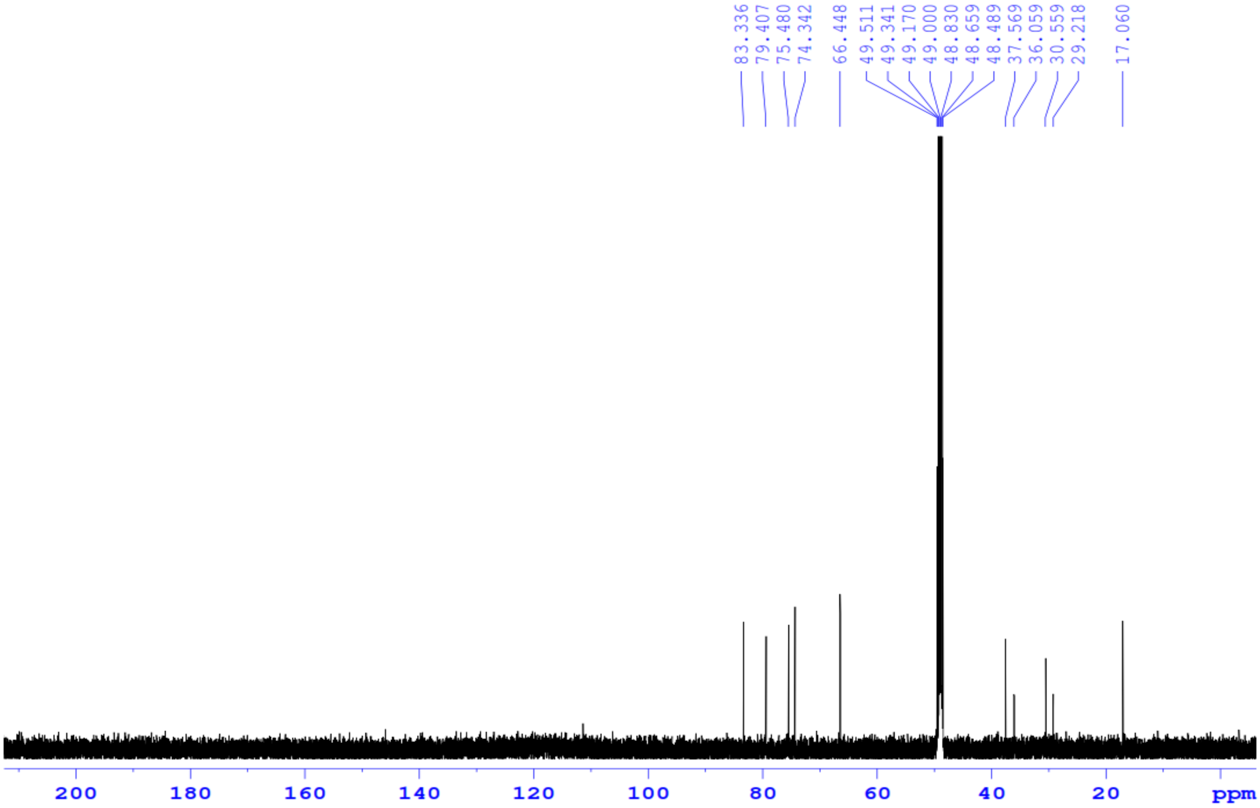


**Figure S11**. $^{13}$C NMR spectrum ($CD_3OD$, 125 MHz) of compound **2**.

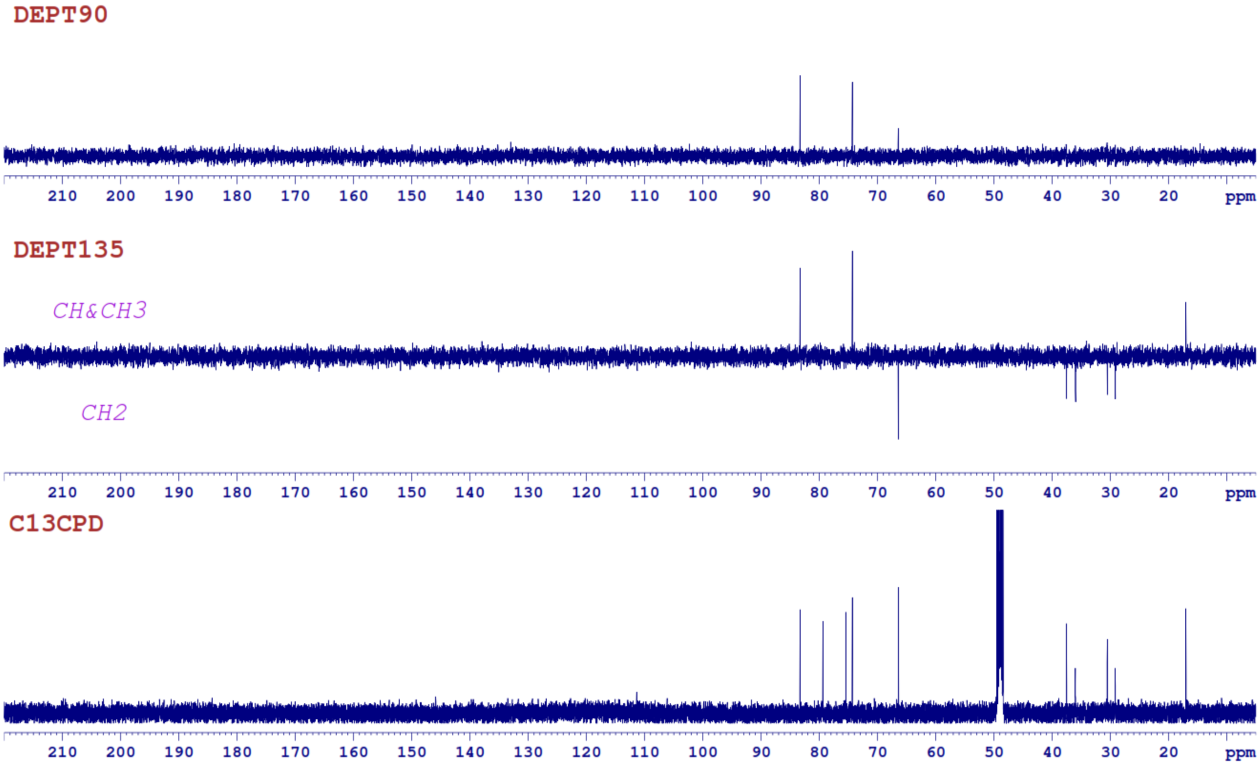


**Figure S12**. DEPT spectrum ($CD_3OD$, 125 MHz) of compound **2**.

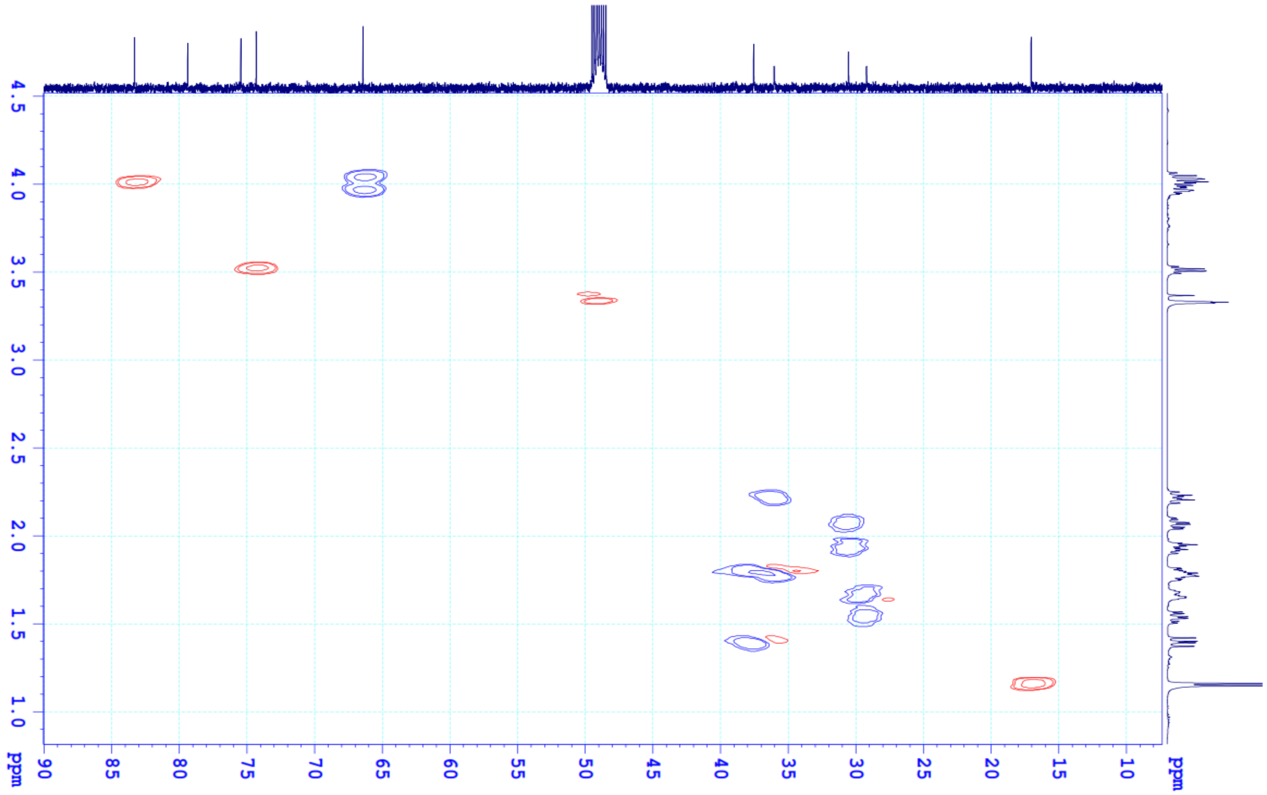


**Figure S13**. HMQC spectrum ($CD_3OD$, 500 MHz) of compound **2**.

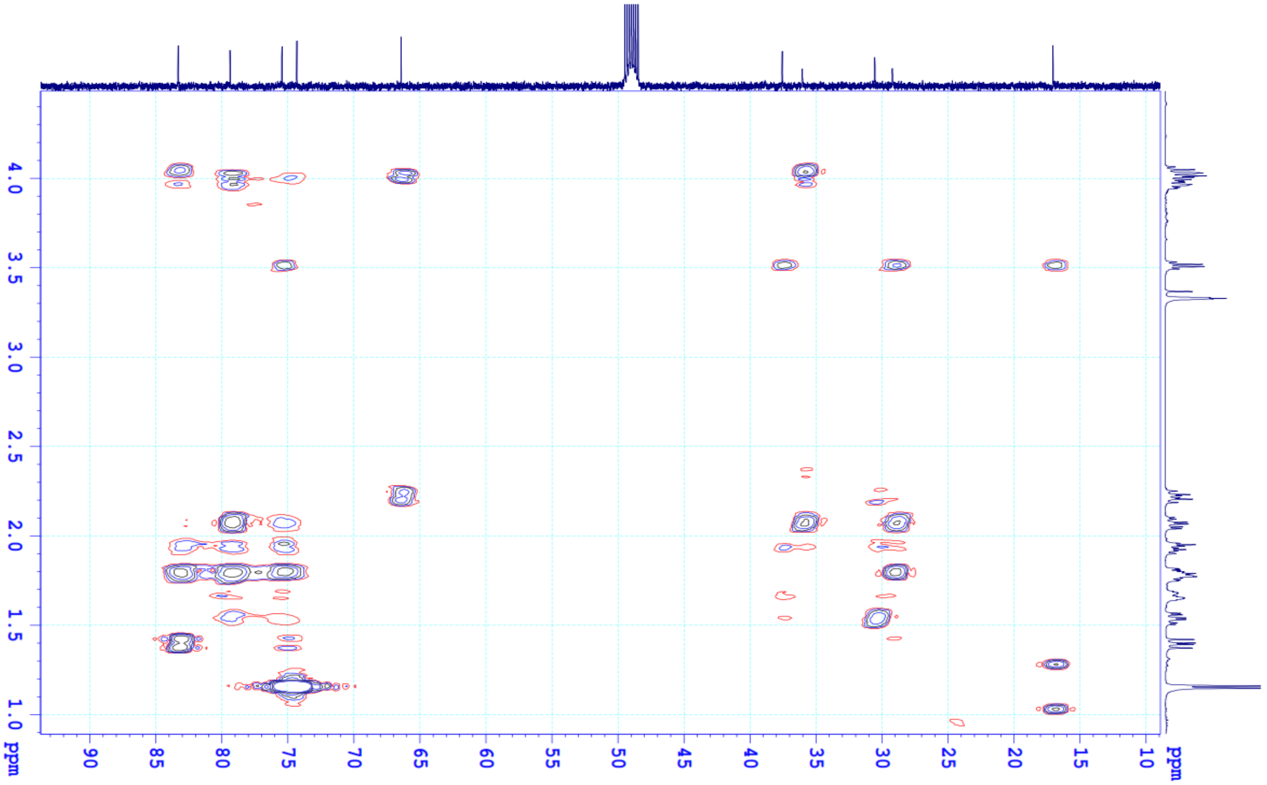


**Figure S14**. HMBC spectrum ($CD_3OD$, 500 MHz) of compound **2**.

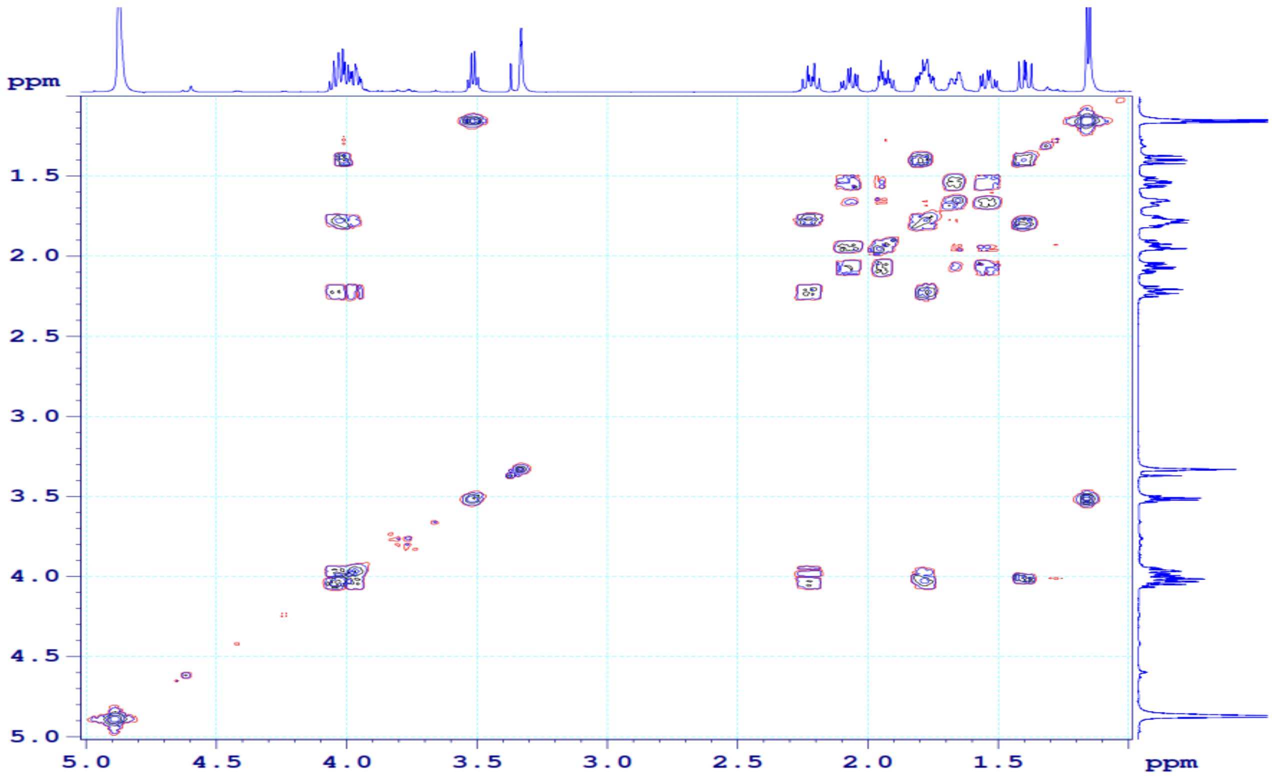


**Figure S15**. COSY spectrum ($CD_3OD$, 500 MHz) of compound **2**.

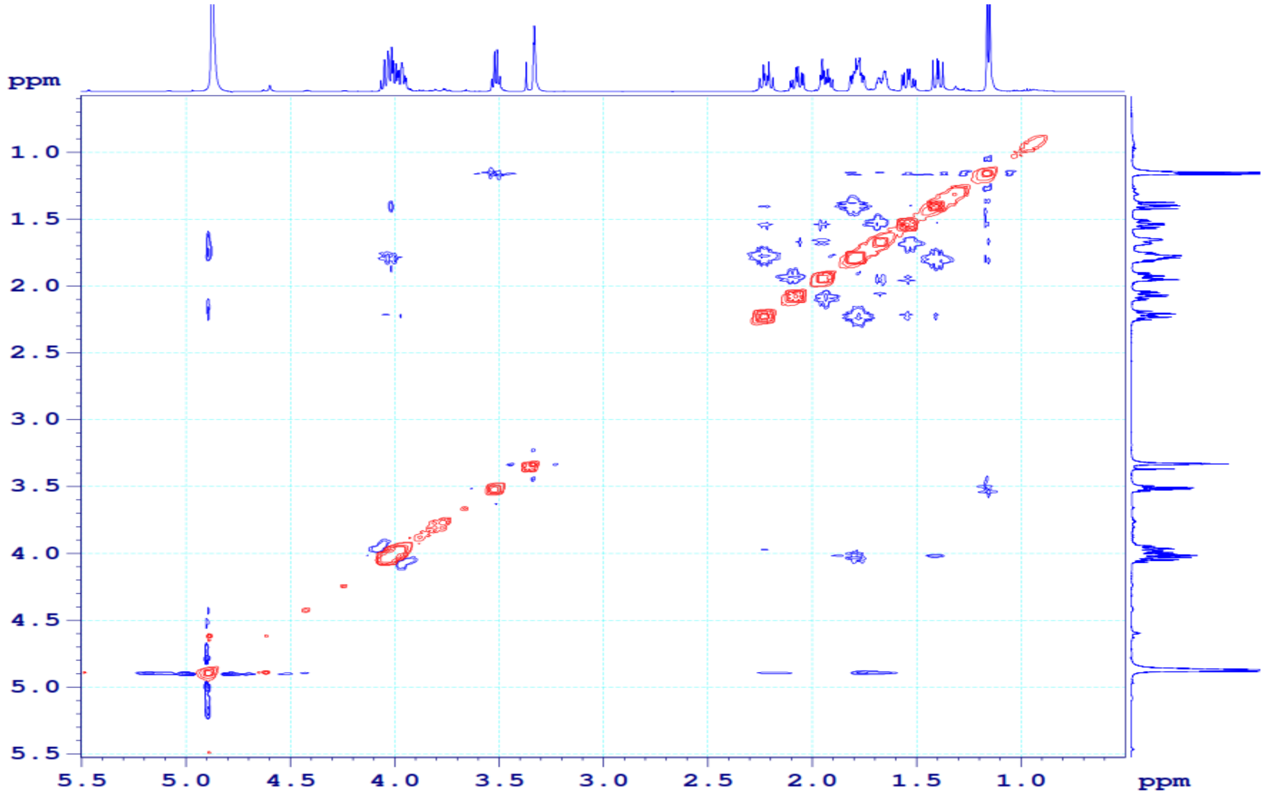


**Figure S16**. NOESY spectrum ($CD_3OD$, 500 MHz) of compound **2**.

## $^{1}$H and $^{13}$C NMR spectroscopic data of isolated compounds 3–5 and 7–12

*Clerindicin E* (**3**): Colorless oil; $[\alpha]_D^{23}$ + 1.15° (*c* 0.011, MeOH); IR (dry film) $\nu_{max}$ 3300, 2950, 1430, 1270, 1170, 920, and 850 cm$^{-1}$; EIMS *m/z* 158 $[M]^+$, 140 $[M - H_2O]^+$, and 122 $[M - 2H_2O]^+$; $^{1}$H NMR (500 MHz, $CD_3OD$): $\delta_H$ 1.97 (2H, m, H-3), 3.85 (2H, m, H-8), 3.93 (1H, m, H-2), and 3.95 (1H, m, H-4). $^{13}$C NMR (125 MHz, $CD_3OD$): $\delta_C$ 75.2 (C-1), 82.1 (C-2), 35.8 (C-3), 66.7 (C-4), 31.0 (C-5), 32.7 (C-6), 39.4 (C-7), and 66.0 (C-8).

*Rengyol* (**4**): White solid; mp 124°C; $[\alpha]_D^{23}$ + 0.8° (*c* 0.01, MeOH); $^{1}$H NMR (500 MHz, $CD_3OD$): $\delta_H$ 1.39 – 1.75 (10H, m, H-2, H-3, H-5, H-6, and H-7), 3.75 (2H, t, *J* = 7.5 Hz, H-1), and 3.54 (1H, broad quintet, *J* = 5.0 Hz, H-1); $^{13}$C NMR (125 MHz, $CD_3OD$): $\delta_C$ 70.7 (C-1), 36.0 (C-2), 31.8 (C-3), 70.7 (C-4), 31.2 (C-5), 36.0 (C-6), 45.5 (C-7), and 59.1 (C-8).

*Cornoside* (**5**): Brown oil; $[\alpha]_D^{23}$ –19.5° (*c* 1.5, EtOH); $^{1}$H NMR (500 MHz, $CD_3OD$): $\delta_H$ 2.07 (2H, t, *J* = 6.5 Hz, H-7), 3.16 – 3.69 (6H, H-2′, H-3′, H-4′, H-5′, and H-6′), 3.87 (1H, dt, *J* = 6.5 Hz, H-8a), 4.01 (1H, dt, *J* = 6.5 Hz, H-8b), 4.24 (1H, d, *J* = 8.0 Hz, H-1′), 6.14 (2H, dd, *J* = 8.0, 1.5 Hz, H-2 and H-6), and 7.04 (2H, dd, *J* = 8.0, 1.5 Hz, H-3 and H-5); $^{13}$C NMR (125 MHz, $CD_3OD$): $\delta_C$ 69.3 (C-1), 154.4 (C-2), 127.9 (C-3), 188.0 (C-4), 127. (C-5), 154.4 (C-6), 41.0 (C-7), 65.7 (C-8), 104.3 (C-1′), 75.0 (C-2′), 78.5 (C-3′), 71.6 (C-4′), 77.9 (C-5′), and 62.7 (C-6′).

*Jaceosidin* (**7**): Pale yellow crystals; mp 259 - 260°C; $^{1}$H NMR (500 MHz, DMSO-$d_6$): $\delta_H$ 3.75 (3H, s, 6-$OCH_3$), 3.88 (3H, s, 3′-$OCH_3$), 6.60 (1H, s, H-8), 6.86 (1H, s, H-3), 7.54 (1H, br s, H-2′); 6.92 (1H, d, *J* = 9.0 Hz, H-5′), and 7.53 (1H, br d, *J* = 9.0 Hz, H-6′); $^{13}$C NMR (125 MHz, DMSO-$d_6$): $\delta_C$ 164.0 (C-2), 103.0 (C-3), 182.4 (C-4), 153.0 (C-5), 131.6 (C-6), 157.5 (C-7), 94.6 (C-8), 152.7 (C-9), 104.4 (C-10), 121.8 (C-1′), 110.4 (C-2′), 148.3 (C-3′), 151.0 (C-4′), 116.0 (C-5′), 120.6 (C-6′), 56.2 (3′-$OCH_3$), and 60.2 (6-$OCH_3$).

*Pomonic acid* (**8**): Crystalline powder; $[\alpha]_D^{23}$ + 50.0° (*c* 0.02, $CHCl_3$); mp 202 - 203°C; $^{1}$H NMR (500 MHz, $CD_3OD$): $\delta_H$ 0.88 (3H, s, H-26), 0.95 (3H, d, *J* = 6.5 Hz, H-30), 1.04 (1H, m, H-

15b), 1.07 (3H, s, H-24), 1.10 (3H, s, H-25), 1.11 (3H, s, H-23), 1.21 (3H, s, H-29), 1.27 (1H, m, H-2b), 1.36 (1H, m, H-20), 1.37 (3H, s, H-27), 1.41 (1H, m, H-5), 1.41 (1H, m, H-7b), 1.48 (1H, m, H-22b), 1.54 (1H, m, H-6), 1.56 (1H, m, H-16b), 1.63 (1H, m, H-7a), 1.64 (1H, m, H-1a), 1.74 (1H, m, H-2a), 1.76 (1H, m, H-1b), 1.82 (1H, m, H-9), 1.84 (1H, m, H-15a), 1.94 (1H, m, H-22a), 2.07 (2H, dd, $J$ = 3.5, 8.0 Hz, H-11), 2.40 (1H, m, H-21b), 2.53 (1H, s, H-18), 2.60 (1H, m, H-16a), 2.62 (1H, m, H-21a), and 5.32 (1H, t, $J$ = 3.5 Hz, H-12); $^{13}$C NMR (125 MHz, $CD_3OD$): $\delta_C$ 38.9 (C-1), 27.2 (C-2), 220.5 (C-3), 48.5 (C-4), 56.6 (C-5), 20.8 (C-6), 33.7 (C-7), 41.0 (C-8), 47.7 (C-9), 37.8 (C-10), 24.7 (C-11), 129.2 (C-12), 140.0 (C-13), 42.7 (C-14), 29.6 (C-15), 26.6 (C-16), 49.2 (C-17), 55.1 (C-18), 73.5 (C-19), 43.0 (C-20), 35.1 (C-21), 40.2 (C-22), 27.0 (C-23), 21.9 (C-24), 15.4 (C-25), 17.3 (C-26), 24.6 (C-27), 182.5 (C-28), 27.1 (C-29), and 16.5 (C-30).

*Pomolic acid* (**9**): Crystalline powder; $[\alpha]_D^{23}$ + 22.6$^o$ ($c$ 0.01, $CHCl_3$); $^1$H NMR (500 MHz, $CD_3OD$): $\delta_H$ 0.81 (3H, s, H-26), 0.86 (3H, s, H-24), 0.94 (3H, d, $J$ = 6.5 Hz, H-30), 0.95 (3H, s, H-23), 0.97 (3H, s, H-25), 1.03 (1H, m, H-15b), 1.21 (3H, s, H-29), 1.26 (1H, m, H-21b), 1.34 (1H, m, H-7b), 1.35 (1H, m, H-5), 1.38 (3H, s, H-27), 1.39 (1H, m, H-20), 1.40 (1H, m, H-1b), 1.42 (1H, m, H-6b), 1.46 (1H, m, H-6a), 1.55 (1H, m, H-2b), 1.64 (1H, m, H-7a), 1.65 (1H, m, H-1a), 1.66 (1H, m, H-22b), 1.72 (1H, m, H-21a), 1.76 (1H, m, H-22a), 1.82 (1H, m, H-15a), 1.88 (1H, m, H-9), 1.98 (1H, m, H-11b), 2.00 (1H, m, H-2a), 2.03 (1H, m, H-11a), 2.52 (1H, s, H-18), 2.60 (2H, m, H-16), 3.36 (1H, d, $J$ = 5.0, H-3), and 5.31 (1H, br t, $J$ = 3.5 Hz, H-12); $^{13}$C NMR (125 MHz, $CD_3OD$): $\delta_C$ 34.2 (C-1), 26.2 (C-2), 76.9 (C-3), 38.3 (C-4), 50.0 (C-5), 19.4 (C-6), 34.1 (C-7), 41.2 (C-8), 48.2 (C-9), 38.1 (C-10), 24.6 (C-11), 129.5 (C-12), 139.9 (C-13), 42.7 (C-14), 29.5 (C-15), 26.6 (C-16), 49.5 (C-17), 55.1 (C-18), 73.6 (C-19), 43.0 (C-20), 27.2 (C-21), 39.0 (C-22), 29.0 (C-23), 22.9 (C-24), 15.7 (C-25), 17.5 (C-26), 24.8 (C-27), 182.5 (C-28), 27.0 (C-29), and 16.5 (C-30).
